# Proceedings Cleveland Dental Society

**Published:** 1887-02

**Authors:** 


### Proceedings Cleveland Dental Society.

The Cleveland Dental Society held its first regular meeting since its organization, at the office of Dr. D. R. Jennings on Saturday afternoon, January 8th; the President, Dr. Jennings in the chair; Dr. J. E. Robinson, Secretary pro tem.

After some miscellaneous business had been disposed of Dr. Stephan was called to the chair and Dr. Jennings read a very interesting paper on "Alveolar Abscess," giving his method of treatment, etc.

The paper was ably discussed by Drs. Barnes.—L. Buffett, Butler, Robinson, Harvey, Owens, and others and much light thrown upon what always has been, and must continue to be, a very interesting subject to the dental profession. Hereafter the meetings will be held at the dental depot of Coggswell & Co. on the first Saturday of each month.

The paper for the next meeting will be furnished by Dr. C. R. Butler; subject, "Simple Tumors of the Mouth including Antrum." Drs. L. Bufiett and J. R Owens will open the discussion.

J. E. Robinson, Sec. pro tem.

